# Catheter ablation of typical atrial flutter in a patient with left ventricular assist device support: a case report

**DOI:** 10.1186/s43044-026-00760-5

**Published:** 2026-06-21

**Authors:** Azmican Kaya, Murat Karaçam, Tezel Kovancı, Seda Tanyeri Uzel, Abdülkadir Uslu

**Affiliations:** 1 Department of Cardiology, Kosuyolu Yuksek Ihtisas Egitim ve Arastirma Hastanesi, Istanbul, Turkey; 2 Department of Cardiology, Bitlis State Hospital, Bitlis, Turkey

**Keywords:** Atrial flutter, Catheter ablation, Left ventricular assist device, Right ventricular function, Heart failure, Case report

## Abstract

**Background:**

Atrial arrhythmias are a frequent and clinically significant complication in left ventricular assist device (LVAD)–supported patients, with the potential to impair right ventricular (RV) function, compromise device hemodynamics, and reduce quality of life. While atrial fibrillation (AF) has received considerable attention in this population, the distinct hemodynamic impact of typical cavotricuspid isthmus (CTI)–dependent atrial flutter and the role of catheter-based rhythm control remain poorly characterized.

**Case presentation:**

A 60-year-old male with non-ischemic cardiomyopathy who underwent HeartMate 3 LVAD implantation as a bridge to transplantation developed typical CTI-dependent atrial flutter refractory to medical therapy, associated with marked deterioration in RV systolic function (TAPSE: 1.2 → 0.82 cm; Sm: 6.9 → 5.3 cm/s) and adverse LVAD hemodynamics. Electrical cardioversion had been attempted but failed to maintain sinus rhythm. Electrophysiological study using three-dimensional electroanatomic mapping (CARTO system, Biosense Webster) confirmed counterclockwise CTI-dependent atrial flutter. Radiofrequency (RF) ablation of the cavotricuspid isthmus achieved bidirectional conduction block and restored sinus rhythm. The procedure was performed under uninterrupted therapeutic anticoagulation with warfarin.

**Results:**

At one-month follow-up, RV systolic function improved substantially (TAPSE: 0.82 → 1.19 cm; Sm: 5.3 → 6.5 cm/s), with concurrent normalization of LVAD hemodynamic parameters and complete resolution of palpitations and dyspnea.

**Conclusions:**

Catheter ablation of CTI-dependent atrial flutter in a carefully selected LVAD-supported patient yielded significant improvement in RV function, device hemodynamics, and quality of life (QoL), underscoring the potential role of rhythm control in this population.

## Introduction

Advanced heart failure management has been transformed by the increasing use of left ventricular assist devices (LVADs) as bridge-to-transplantation or destination therapy, with demonstrated improvements in survival and functional status [1]. Atrial arrhythmias complicate the clinical course of LVAD-supported patients in 21–54% of cases, impairing atrioventricular (AV) synchrony, reducing RV filling, and compromising preload-dependent device hemodynamics [2, 3]. Despite this high prevalence, evidence guiding optimal management remains limited—particularly with regard to catheter-based rhythm control. Typical CTI-dependent atrial flutter represents a distinct electrophysiological entity from atrial fibrillation (AF), with an anatomically targetable substrate and high procedural success rates that make it particularly amenable to ablation. We present a case of successful CTI ablation in a HeartMate 3 LVAD recipient, with objective documentation of RV functional recovery and hemodynamic improvement.

## Case presentation

A 60-year-old male with non-ischemic cardiomyopathy underwent implantation of a HeartMate 3 LVAD (Abbott, Chicago, IL, USA) as a bridge to cardiac transplantation, having remained symptomatic despite optimal guideline-directed medical and device therapy. The postoperative course was uneventful, and the patient remained clinically stable and asymptomatic during routine follow-up. He subsequently presented with new-onset palpitations and progressive exertional dyspnea, consistent with New York Heart Association (NYHA) class III functional status.

His relevant medical history included prior implantation of a cardiac resynchronization therapy defibrillator (CRT-D), electrical cardioversion, and pulmonary vein isolation for AF—all performed before LVAD implantation. His current pharmacological regimen comprised ramipril, metoprolol, spironolactone, empagliflozin, amiodarone, furosemide, and warfarin.

**Fig. 1:**
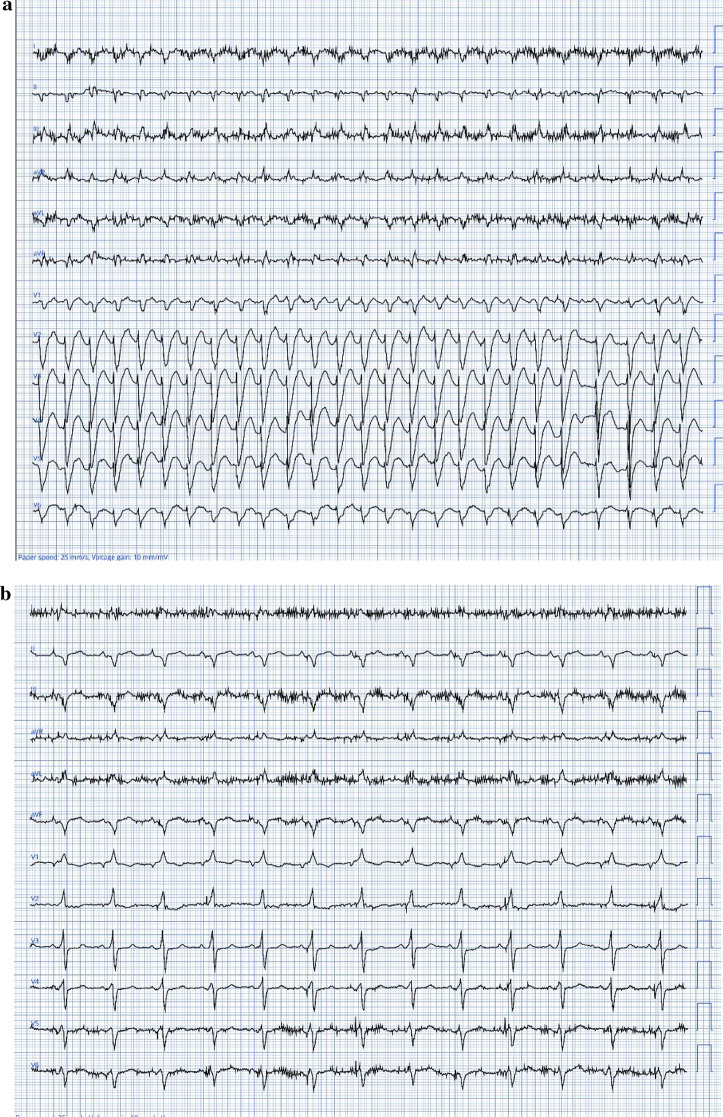
**a** Twelve-lead electrocardiogram demonstrating typical atrial flutter with 2:1 atrioventricular conduction and a wide QRS complex, at a ventricular rate of 140–150 beats per minute. Periodic low-frequency EMI artifacts, characteristic of continuous-flow LVAD operation, are visible across multiple leads and should be recognized to avoid misinterpretation as additional arrhythmia or noise. **b** Twelve-lead electrocardiogram following successful RF ablation of the cavotricuspid isthmus, demonstrating restoration of sinus rhythm. Residual LVAD-related EMI artifacts remain visible, confirming ongoing device operation

**Fig. 2:**
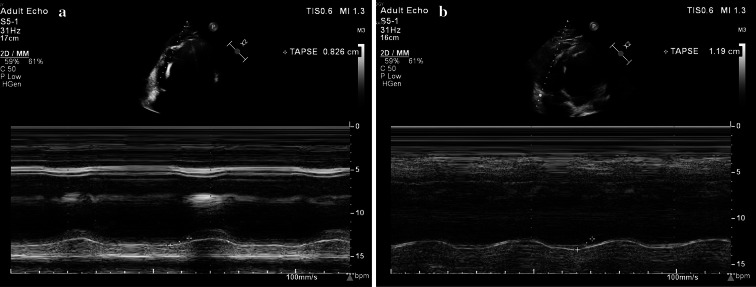
**a** TTE performed during atrial flutter, demonstrating significant RV systolic dysfunction (TAPSE 0.82 cm; Sm 5.3 cm/s). **b**. TTE at one-month follow-up following successful catheter ablation, demonstrating marked improvement in RV systolic function (TAPSE 1.19 cm; Sm 6.5 cm/s)

On presentation, the patient was hemodynamically stable. Surface 12-lead electrocardiography (ECG) revealed typical atrial flutter with 2:1 atrioventricular conduction and a wide QRS complex, at ventricular rates of 140–150 beats per minute (Fig. 1a). Periodic low-frequency electromagnetic interference (EMI) artifacts, characteristic of continuous-flow LVAD operation, were visible across multiple leads and should be recognized to avoid misinterpretation as additional arrhythmia or noise. For reference, transthoracic echocardiography (TTE) performed in sinus rhythm prior to the index arrhythmia had documented relatively preserved RV systolic function (TAPSE 1.2 cm; Sm 6.9 cm/s). Repeat TTE during atrial flutter demonstrated significant deterioration, with TAPSE declining to 0.82 cm and Sm to 5.3 cm/s (Fig. 2a). Concurrent LVAD-related hemodynamic compromise was evident, manifesting as persistent aortic valve closure and leftward interventricular septal displacement consistent with suction events.

Electrical cardioversion had been attempted prior to referral for ablation; however, sustained sinus rhythm could not be maintained. Rate control with metoprolol and amiodarone subsequently proved inadequate. In view of the progressive RV dysfunction, adverse LVAD hemodynamics, and refractory symptom burden, a decision was made to proceed with catheter ablation.

Prior to the procedure, transesophageal echocardiography (TEE) was not performed, as the patient was maintained on uninterrupted therapeutic anticoagulation with warfarin throughout, consistent with the high thromboembolic risk inherent to LVAD-supported patients.

**Fig. 3:**
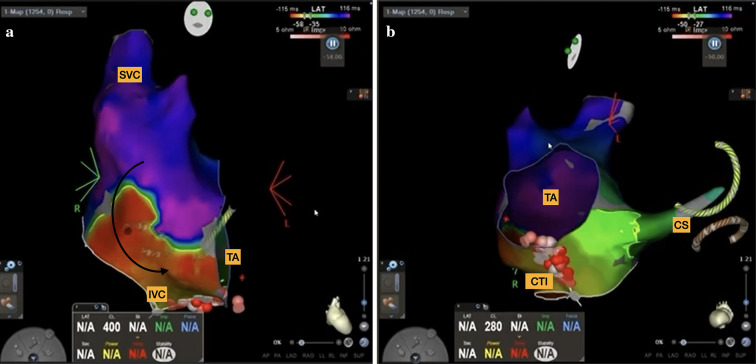
**a** Three-dimensional electroanatomic LAT (local activation time) map (CARTO system, Biosense Webster) confirming typical counterclockwise CTI-dependent atrial flutter. The black arrow indicates the direction of activation wavefront propagation around the tricuspid annulus (TA). SVC: superior vena cava; IVC: inferior vena cava; TA: tricuspid annulus. **b** Electroanatomic map demonstrating the RF ablation line across the cavotricuspid isthmus. Ablation tag represent radiofrequency energy application points along the CTI. TA: tricuspid annulus; CS: coronary sinus; CTI: cavotricuspid isthmus

**Fig. 4:**
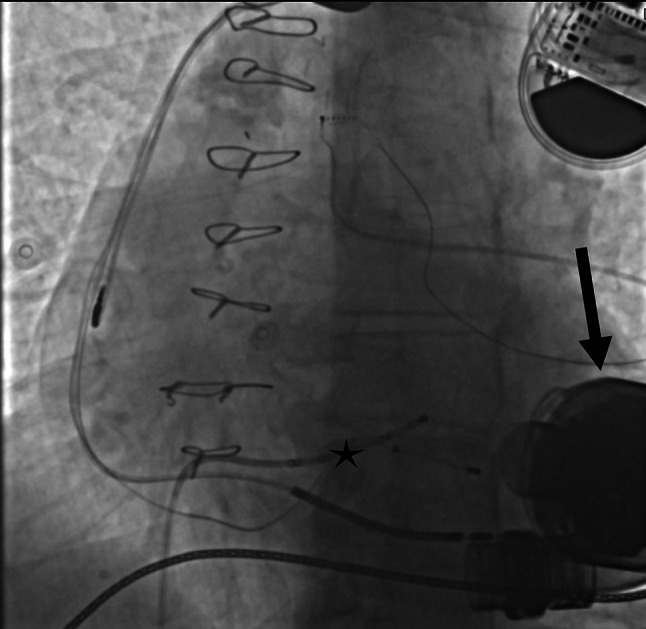
Fluoroscopic image obtained during catheter ablation. The star (★) indicates the position of the coronary sinus (CS) catheter. The arrow indicates the LVAD pump, highlighting the importance of fluoroscopic guidance in the context of altered cardiac anatomy in LVAD-supported patients

Electrophysiological study was performed under three-dimensional electroanatomic guidance (CARTO system, Biosense Webster). Activation and entrainment mapping confirmed typical counterclockwise CTI-dependent atrial flutter (Fig. 3a, b). Radiofrequency (RF) energy delivery across the cavotricuspid isthmus achieved bidirectional conduction block and restored sinus rhythm acutely (Fig. 1b). Fluoroscopic images during the procedure are shown in Fig. 4.

Early post-procedural TTE demonstrated stabilization of RV systolic function. At one-month follow-up, marked improvement was observed in RV parameters (TAPSE 1.19 cm; Sm 6.5 cm/s; Fig. 2b), along with normalization of LVAD hemodynamics: the aortic valve exhibited intermittent opening every 2–3 cardiac cycles, and the interventricular septum returned to a midline position. Clinically, the patient reported complete resolution of palpitations and dyspnea, with substantial improvement in exercise capacity and QoL Fig. 4.

## Discussion

Atrial arrhythmias occur in 21–54% of LVAD-supported patients, with new-onset arrhythmias developing in approximately 20–30% following implantation [4]. Despite advances in continuous-flow, magnetically levitated LVAD technology, right heart failure remains a principal determinant of morbidity and mortality in this population [5, 6]. RV dysfunction following LVAD implantation is multifactorial; atrial arrhythmias exacerbate this vulnerability by impairing AV synchrony, reducing RV filling, and compromising the preload-dependent hemodynamics that underpin effective LVAD function [3].

Evidence guiding rhythm control in LVAD recipients is heterogeneous and largely derived from retrospective, single-center experiences. While catheter ablation is well established as an effective rhythm control strategy in the broader heart failure population, its role in LVAD-supported patients has not been clearly defined [7]. Prior reports have yielded mixed findings. A small case series of HeartMate 2 recipients undergoing ablation for atrial flutter demonstrated improvements in functional status and symptom burden [8], and isolated reports of HeartMate 3 recipients have described hemodynamic improvement following ablation [9]. Conversely, a larger retrospective analysis of 418 LVAD patients found no significant association between rhythm control strategy and clinical outcomes [2]. These discrepant findings likely reflect the heterogeneity of arrhythmia type and substrate, patient selection, and the predominant reliance on pharmacological rather than catheter-based approaches.

The clinical impact of typical atrial flutter in LVAD-supported patients warrants specific consideration, distinct from that of AF. The fixed, rapid atrial cycle length characteristic of CTI-dependent flutter produces loss of AV synchrony and impaired RV diastolic filling—hemodynamic perturbations that may be poorly tolerated in patients with diminished RV reserve. In the present case, atrial flutter precipitated marked deterioration in RV systolic indices and LVAD performance, both of which recovered substantially following successful ablation. The concordance between echocardiographic improvement and complete clinical resolution reinforces the mechanistic link between rhythm control, RV-LVAD coupling, and patient-centered outcomes.

Several procedural considerations are particularly relevant when performing catheter ablation in LVAD-supported patients. EMI generated by the LVAD motor may affect intracardiac electrogram quality and surface ECG interpretation; recognition of these artifacts is essential for accurate mapping and ablation. Catheter stability may be compromised due to altered cardiac geometry and reduced intracavitary blood volume secondary to continuous-flow unloading; the use of long vascular sheaths may improve catheter contact force and stability at the ablation site. Anticoagulation management is a critical periprocedural consideration: in the present case, warfarin was continued without interruption, maintaining therapeutic anticoagulation throughout, consistent with the high thromboembolic risk in LVAD-supported patients. Fluoroscopic guidance remains a valuable adjunct in this population to confirm catheter positioning in the context of altered cardiac anatomy [3].

Typical CTI-dependent atrial flutter represents a well-characterized, anatomically targetable substrate with high procedural success rates and low recurrence following ablation—a favorable profile that distinguishes it from the more complex substrate of AF. In selected LVAD-supported patients with typical atrial flutter and evidence of hemodynamic compromise, catheter ablation may therefore offer a clinically meaningful and durable therapeutic option that pharmacological rate or rhythm control cannot reliably provide.

## Conclusion

This case demonstrates that catheter ablation of CTI-dependent atrial flutter in a carefully selected LVAD-supported patient can yield significant improvement in RV systolic function, LVAD hemodynamics, and QoL. These findings support heightened clinical consideration of catheter-based rhythm control in LVAD recipients presenting with typical atrial flutter and hemodynamic compromise. Prospective multicenter studies are needed to establish the safety, efficacy, and long-term impact of this approach in the broader LVAD population.

## Data Availability

No datasets were generated or analysed during the current study.

## Abbreviations

AF: Atrial Fibrillation
AV: Atrioventricular
CRT-D: Cardiac Resynchronization Therapy Defibrillator
CTI: Cavotricuspid Isthmus
EMI: Electromagnetic Interference
LVAD: Left Ventricular Assist Device
NYHA: New York Heart Association
QoL: Quality of Life
RF: Radiofrequency
RV: Right Ventricle / Right Ventricular
Sm: Systolic Myocardial Velocity (tissue Doppler imaging)
TAPSE: Tricuspid Annular Plane Systolic Excursion
TEE: Transesophageal Echocardiography
TTE: Transthoracic Echocardiography
